# So Fragile, So Human: Noncoding DNA Regions Orchestrating Gene Expression Involved in Neurodevelopmental Disorders and in Human Brain Evolution

**DOI:** 10.3390/ijms27062785

**Published:** 2026-03-19

**Authors:** Carolina Marenco, Giorgia Pozzolini, Martina Casciaro, Matheo Morales, Cristiana Barone, Delia Morciano, Cristian Barillari, Elvira Zakirova, Gabriele Antoniazzi, Theresa Lahoud, Filippo Mosconi, Davide Cabassi, James P. Noonan, Elena Bacchelli, Silvia K. Nicolis

**Affiliations:** 1 Dipartimento di Biotecnologie e Bioscienze, Università di Milano-Bicocca, 20126 Milano, Italy; carolina.tide@hotmail.it (C.M.); g.pozzolini@campus.unimib.it (G.P.); cristiana.barone@unimib.it (C.B.); d.morciano@campus.unimib.it (D.M.); c.barillari1@campus.unimib.it (C.B.); elvira.zakirova@unimib.it (E.Z.); posta.antoniazzigabriele@gmail.com (G.A.); theresa.lahoud@net.usj.edu.lb (T.L.); f.mosconi4@campus.unimib.it (F.M.); 2 Dipartimento di Farmacia e Biotecnologie, Università di Bologna, 40126 Bologna, Italy; martina.casciaro2@unibo.it (M.C.); elena.bacchelli@unibo.it (E.B.); 3 Department of Genetics, Yale School of Medicine, New Haven, CT 06510, USA; matheo.morales@yale.edu (M.M.); james.noonan@yale.edu (J.P.N.); 4 Conservatorio Luca Marenzio, 25121 Brescia, Italy; davidecabassi@gmail.com

**Keywords:** gene regulation, gene regulatory networks, transcription factors, SOX2, neurodevelopmental disorders, nervous system, brain development, autism

## Abstract

The development of the human brain starts with the orchestrated expression of our genes during embryogenesis. Non-protein-coding DNA sequences (gene promoters and enhancers) dynamically interact to form a three-dimensional (3D) network, orchestrating gene expression. We discuss novel perspectives on how DNA sequence variants within regulatory DNA, identified by whole-genome sequencing (WGS), contribute to the development of neurodevelopmental disorders (NDDs), including autism spectrum disorders (ASDs). We discuss two recent models explaining the evolution of a subset of regulatory sequences, Human Accelerated DNA Regions (HARs), proposed to be involved in the evolution of uniquely human brain features through their participation in the 3D interactions network. We connect this with the recent proposal that rare, recessive inherited sequence variants within HARs, interacting with distant target genes in neural cells, represent risk factors for the development of ASDs. The SOX2 transcription factor, whose heterozygous mutation causes NDDs, shapes the noncoding-DNA interaction network in neural cells, and binds DNA together with FOS, whose recognition sequence is enriched within HARs carrying human-specific substitutions modulating enhancer activity. SOX2 also binds regulatory regions (including HARs) carrying ASD-associated mutations. We highlight research directions based on these findings, which will hopefully improve our understanding of the connection between SOX2-dependent gene regulatory networks, NDDs, and brain evolution.

## 1. Introduction

**Figure 1:**
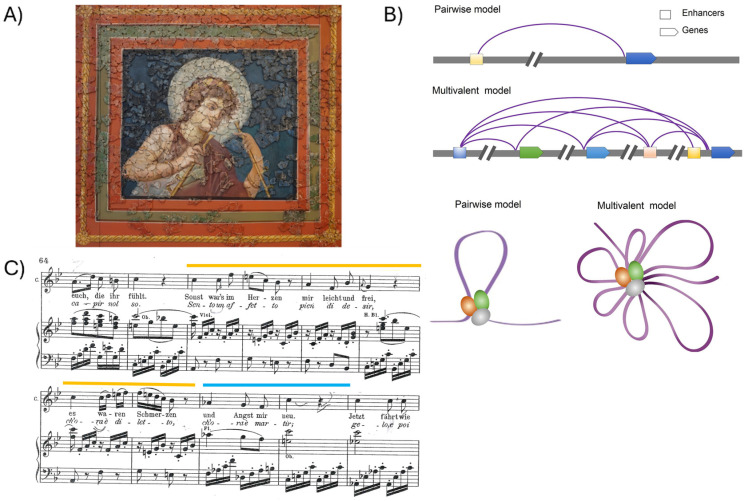
Emerging properties. (**A**) The joyful, attentive attitude of the musician about to start playing emerges from the composition of thousands of fragments, precisely joined to one another. This ancient Roman painting was found as a heap of tiny fragments under the Trier Cathedral (Germany), and patiently recomposed by archaeologists and their students over many years (now in the Cathedral Museum, Trier, Germany. Image Copyright: Roman ceiling painting, 4th century, © M. Groß-Morgen, Museum am Dom Trier). Note how specific fragments, such as those composing the smile, and the eyes, are especially important “hubs” for our perception of the emotional significance of the global picture. The human mind emerges from the sum and precise interaction of billions of neurons, whose features, numerosity, and interaction patterns at birth are encoded in our genome and develop during embryogenesis. (**B**) At the molecular level, gene expression patterns giving rise to brain development during embryogenesis are orchestrated by a network of three-dimensional (3D) interactions involving protein-coding genes and noncoding, regulatory DNA (promoters, enhancers; see Figure 2 and Figure 3), regulating and finely tuning gene transcription in neural cells. Genome-wide studies of enhancer–promoter connectivity showed that one promoter is generally connected to more than one enhancer, and vice versa, according to a multivalent (rather than simply pairwise) model of interactivity (modified from [3,4]). Postnatal development of the brain and mind will build onto this network, through interaction with the environment; this can reconfigure aspects of the network acting through critical periods, epigenetics, and experience-dependent plasticity [5,6]. (**C**) In music, in a way similarly, emotional significance emerges from the interaction between notes. For example, the major and minor modes make use of different scale types, differentiated by the intervals between notes, providing different emotional qualities to music [7,8] (see also Section 10). In the example shown (W.A. Mozart, Le Nozze di Figaro, act II, Cherubino, Canzone), Mozart switches (modulates) from major (yellow) to minor (blue) mode within the same musical phrase (The A-flat note at the beginning of the blue bit is essential for the switch to the minor mode; in the major mode, this would be an A). Interactions between notes are both “vertical” (simultaneous notes forming chords) and “horizontal” (connections between chords, or individual notes, forming musical phrases; see Section 10).

At every new generation, two copies of the human genome, one maternal and one paternal, contained within a single cell, the fertilized egg, generate a new human being, equipped with a brain that will think, imagine, decide, drive actions, and dream. The primary mechanism underlying this is the ordered, regulated activation of gene expression programs during embryonic development, leading to the formation of a brain constituted of approximately 86 billion neurons [1], each interconnected with many others in a network whose characteristics at birth are encoded in our DNA. This emerges from the interplay of “protein-coding” DNA and “non-coding” DNA elements, guided by specialized proteins (transcription factors) and regulatory RNAs, responsible for the regulation and fine-tuning of gene expression in space and time. The complexity of the brain and its functions “emerge” as a global property from the many molecular interactions between DNA sequences giving rise to gene expression patterns during development, which in turn shape the network of interactions between neurons, as a characteristic not present in the individual components, and potentially not entirely predictable from their individual properties [2] (Figure 1).

**Figure 2:**
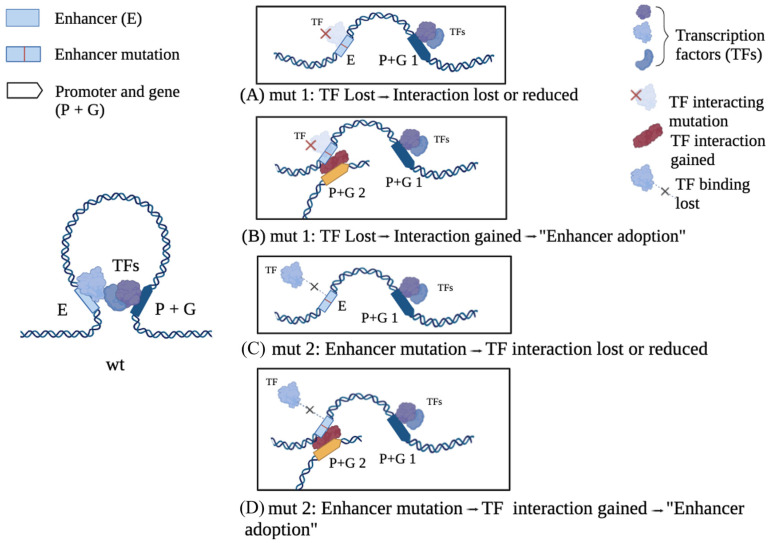
Orchestration of gene expression by regulatory DNA and transcription factors and effects of their mutation. Left: E (enhancer) and P (promoter) are noncoding, regulatory DNA elements that together orchestrate the expression of gene G by binding to specific specialized proteins, transcription factors (TFs), that control gene transcription into RNA. E and P are located far away on the linear DNA (chromosome) map, but TFs connect them physically and functionally to form dynamic loops, termed long-range interactions (LRI). Such complexes accurately regulate gene activity (transcription into RNA, subsequently translated into protein) to occur in specific cell types and developmental stages. LRI between noncoding DNA regions (P, E) are increasingly recognized as a key molecular mechanism for regulated gene expression in complex multicellular organisms such as humans. Right, mut 1 (top two drawings, **A** and **B**) and mut 2 (lower two drawings, **C** and **D**): Regulatory mutations can affect genes encoding for TFs (mut 1), or the regulatory DNA to which a TF binds (mut 2). Both can cause loss of interactions (**A**, **C**), but also gain of novel interactions, by the “adoption” of different enhancers (**B**, **D**). For regulatory DNA and TF control of LRI, in their intersection with genetic disease and human-specific evolution, see in particular [9,10,11].

In this perspective, we recapitulate some recent views on the central role of 3D chromatin organization in transcription (Section 2) and focus on recent models relating DNA sequence variants to the inheritance of neurodevelopmental disorders (NDDs) and psychiatric traits (Section 3). We report on recent findings regarding the genetic association of DNA sequence variants located within transcriptional regulatory elements to autism spectrum disorders (ASDs) (Section 4). We introduce a special subset of regulatory DNA elements, Human Accelerated Regions (Section 5), and present two recent models on their evolution (Section 6). We relate this to the function of SOX2, a transcription factor whose heterozygous mutation causes NDDs, by commenting on SOX2 function in the structuring of the 3D enhancer–promoter interaction network in neural cells, and the involvement of the interacting HAR regulatory elements in NDDs (Section 7). We discuss how mutations within HARs have been involved in ASDs, and how SOX2 is one of the TF whose recognition sequence is most enriched within these HARs (Section 8). Finally, we present some future directions for experimentation suggested by these observations (Section 9). In the final Box (Section 10), we draw a parallel with music, whereby significance emerges from the orchestrated relations between components (DNA sequences; notes and chords).

## 2. Orchestration of Gene Expression by the 3D Organization of Regulatory DNA and Transcription Factors and Effects of Their Mutation

Genes encoding proteins via the genetic code represent about 2% of our DNA. Within the remaining 98%, noncoding, “regulatory” DNA is located, which functions in regulating the transcription of genes, eventually producing fine-tuned levels of mRNAs in a cell-type-specific, time-regulated way to be translated into proteins. The development of efficient DNA sequencing technologies at the genome-wide scale led to the discovery that regulated gene activity is associated with long-range interactions between noncoding DNA sequences, promoted and maintained by transcription factors (TFs), specialized proteins recognizing specific short DNA sequence stretches on DNA (Figure 1B and Figure 2) [9,10,11]. At the genome-wide level, such interactions are structured mostly as a network, in which one given sequence interacts with more than one sequence at any given time (Figure 1B; see [3,4] for specific examples relevant for human NDDs). Sequence variants (single-nucleotide variants (SNVs), indels, and structural variants (SVs)) within regulatory DNA sequences, as well as within genes encoding TFs, can lead to differences in gene transcription that can be relevant for brain development (Figure 2). SNVs may entail the loss of regulatory interactions, as seen for example with point mutations in the distant SIMO enhancer of the PAX6 gene [12]; it may also involve the acquirement of new interactions, as seen with “enhancer adoption” (Figure 2; examples in Figure 6B) [9,10,11].

The sequencing of thousands of human genomes among “healthy” individuals (https://www.internationalgenome.org/, accessed on 15 January 2026; see also gnmAD, https://gnomad.broadinstitute.org/about, containing data from more than 76,000 genomes, accessed on 15 January 2026) as well as individuals affected by neurodevelopmental disorders (NDDs), led to an appreciation of the widespread presence of DNA sequence variants within the noncoding DNA of both groups, potentially affecting gene expression.

## 3. DNA Variants and Models of Inheritance of Neurodevelopmental Disorders and Psychiatric Traits

**Figure 3:**
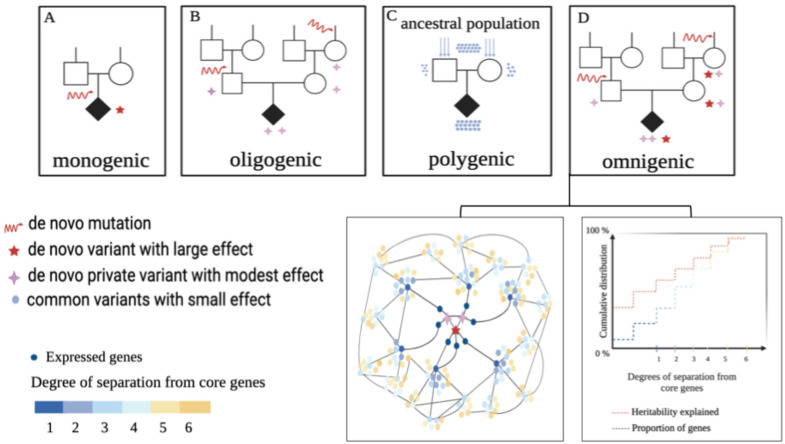
Models of inheritance of neurodevelopmental disorders and traits (adapted from [13,14]. Plausibly, many diversified mind characteristics observed among “normal” individuals are also similarly inherited. (**A**) In rare cases (less than 1% of autism spectrum disorders), a neurodevelopmental disorder can arise as a consequence of a single highly penetrant de novo mutation, hitting the germ cell that will generate the patient; this mutation accounts for most of the disease predisposition (e.g., FOXG1 or TCF4 transcription factor gene mutations, or SHANK or NEUROLIGIN synaptic protein mutations; see text). This inheritance is termed monogenic. (**B**) Two or more DNA sequence variants (mutations), individually of modest effect, arising separately in different individuals, may lead to disease when brought together into one individual by breeding. This inheritance is called oligogenic [14]. (**C**) Many “common” variants, inherited by the patient’s parents from an ancestral population, may lead to high disease predisposition if present in specific combinations, with each of them making a very small contribution (e.g., [16]). This inheritance is termed polygenic. (**D**) In the omnigenic model [13,17,18], variants affecting “core genes” (red and pink stars) propagate their effects through regulatory networks that connect them to many “peripheral genes” influenced by common small-effect variants (blue dots).

NDDs such as autism spectrum disorders (ASDs) and psychiatric disorders such as schizophrenia, major depression, and bipolar disorder are now recognized to have a strong genetic component, involving contributions from both de novo and inherited DNA sequence variation. Their mode of inheritance involves the contribution of DNA sequence variants ranging from rare to common [13,14] (Figure 3). The composite modes of inheritance of NDDs can be exemplified by ASDs. ASDs have an overall frequency of about 1% in human populations, and range from mild conditions, compatible with normal lives and sometimes characterized by exceptional abilities in specific fields, to severe conditions, involving inability to communicate and progressive neural degeneration that can be fatal at young age [15]. At one extreme, rare forms of ASDs can arise as a consequence of a de novo highly penetrant single-gene mutation (monogenic model, Figure 3A); at the other extreme, combinations of “common variants” [16], individually present also in non-affected individuals, can reach a threshold leading to the disorder (polygenic model, Figure 3C). The importance of “common mutations” adding up as small changes collectively leading to a threshold was recently underscored by findings indicating that the susceptibility to rare NDDs is distributed normally in the population, with individuals beyond a given threshold becoming affected (see also Section 4).

Between these two extremes lies a wide range of intermediate genetic architectures. These intermediate scenarios include, for instance, cases where each parent carries rare variants of modest effect that, when co-inherited by the proband, exceed the liability threshold (oligogenic model, Figure 3B).

In many individuals, rare and common variants co-occur and interact, such that neither class of variant would be sufficient, but together they raise liability beyond the diagnostic threshold. Variants of modest or large effect that alter the activity of key “core genes” (red and pink stars in Figure 3D) are embedded within regulatory networks that extend to numerous “peripheral genes” influenced by common, small-effect variants (Figure 3D). This omnigenic model [13,17,18] posits that, through successive layers of regulatory interactions, virtually the entire active genome becomes functionally connected to core genes, such that perturbations to core-gene expression can propagate across the network. Such combinations illustrate how monogenic, oligogenic, polygenic, and omnigenic mechanisms blend along a continuum to shape ASD risk.

The contribution to ASDs of mutations impacting protein-coding genes is well established [15]; the study of rare forms in which a single gene mutation is responsible for most of the pathology first highlighted the contribution of genes encoding proteins active at neuronal synapses, as well as genes encoding TFs, often regulating the former [19,20]. The contribution of mutations affecting regulatory DNA sequences, plausibly smaller for a given mutation, is more difficult to reveal. Furthermore, while mutations in protein-coding genes have effects predictable based on the genetic code, the effects of mutations within regulatory elements are more difficult to ascertain.

## 4. De Novo Noncoding Mutations Associated with Autism Spectrum Disorders Are Located Within Conserved Regulatory DNA Sequences

**Figure 4:**
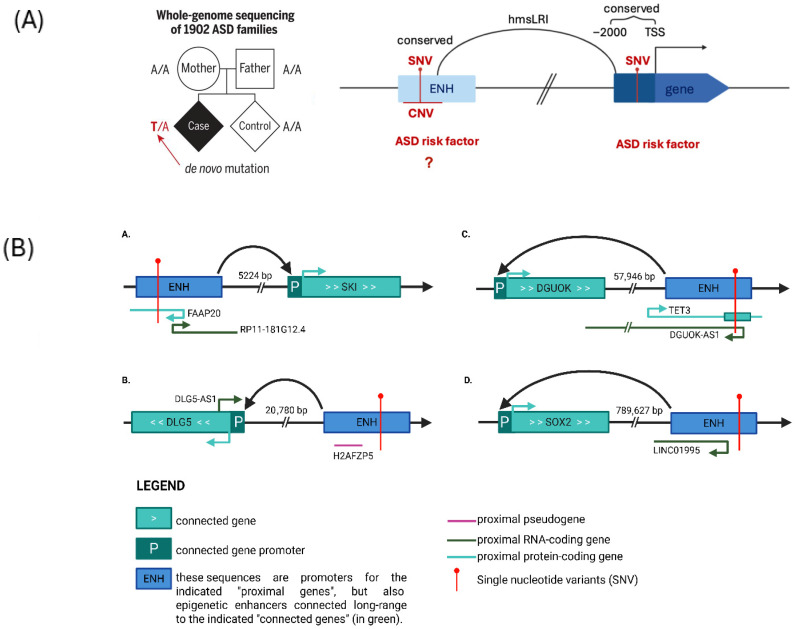
De novo mutations associated with autism spectrum disorders are located within noncoding, regulatory DNA sequences, possibly modulating gene activity. (**A**) Left: Families studied by [21] (adapted from [21]). Right: De novo mutations in promoters and enhancers depicted as “risk factors” because a significant enrichment in de novo mutations in ASD cases versus controls is detected (see text) (i) within promoter regions; this was shown by [21]; (ii) within enhancers involved in human-mouse syntenic long-range interactions (hmsLRI) tethered by RNApolII; this is hypothesized (question mark) based on our preliminary results (E.B. et al. in preparation, and Bacchelli et al. poster P11.001.D in [23]). (**B**) Location of some mutations from [21] within promoters, connected to other promoters via ChIA-PET-mapped interactions from [3,22]. A–D represent four different cases. Such sequences, identified in An et al. as gene promoters, are called “ENH” in the present drawings, in reference to their involvement in interactions with other promoters, onto which they may act as enhancers (and vice versa). Of note, among these connected genes is SOX2 (D), a global regulator of chromatin connectivity and a target of rare, ASD-associated recessive mutations (see Figure 4 and Figure 7).

The identification of noncoding DNA mutations at the genome-wide scale requires efforts in whole-genome sequencing (WGS) and statistical analyses performed on large cohorts of patients. Recently, within the PsychENCODE framework, this challenge was addressed by An et al. [21], who performed WGS on 1902 families from the Simons Simplex Collection (SSC, https://www.sfari.org/resource/simons-simplex-collection/, accessed on 15 December 2025), each including an ASD proband, an unaffected sibling, and one their parents (Figure 4A). In these families, they identified ~67 de novo variants (DNVs) per child, consistent with expected mutation rates. Using machine learning approaches, they developed a de novo risk score to test whether specific categories of DNVs were enriched in ASD cases relative to their unaffected siblings. This analysis revealed a strong contribution to ASD risk from coding DNVs and a weaker, but statistically significant, contribution from noncoding DNVs, particularly within promoter regions, defined as the 2 kb upstream of the transcription start site (TSS) (Figure 4A, right; the An et al. data [21] refer to the promoter region). Interestingly, this promoter signal was enriched specifically in evolutionarily conserved promoters (PhastCons ≥ 0.2 and/or PhyloP ≥ 2), supporting the contribution of noncoding DNVs to ASD risk [21]. Therefore, we hypothesized that conserved enhancers, which form long-range interactions with promoters in neural cells (hmsLRI regions) [3,22], might similarly show enrichment for DNVs in ASD (Figure 4A, right; enhancer region ENH).

To explore this hypothesis, we analyzed the same 1902 SSC quartet families, which allow a direct comparison of ASD probands with their unaffected siblings, while controlling for parental background. Using logistic regression with parental ages at the birth of the child as covariates (because these are known factors influencing the DNV rates—[24]) we assessed whether evolutionarily conserved DNVs (470 mammals PhastCons ≥ 0.2 and/or PhyloP ≥ 0.2) within hmsLRI regions were associated with ASDs. We observed a significant enrichment of conserved DNVs in ASD probands compared to their unaffected siblings (102 vs. 70 DNVs, *p* = 0.014, OR = 1.47), supporting the hypothesis that regulatory DNVs within hmsLRI may contribute to ASD susceptibility (Bacchelli et al. P11.001.D in [23] and Bacchelli et al., manuscript in preparation). Several promoters enriched for DNVs identified by An et al. are involved in long-range interactions with additional promoters, and they may function as enhancers in these contexts (Figure 4B). Interestingly, among them is SOX2, encoding a TF involved in NDDs and essential for the maintenance of the 3D long-range interactions network in NSC (see below).

**Figure 5:**
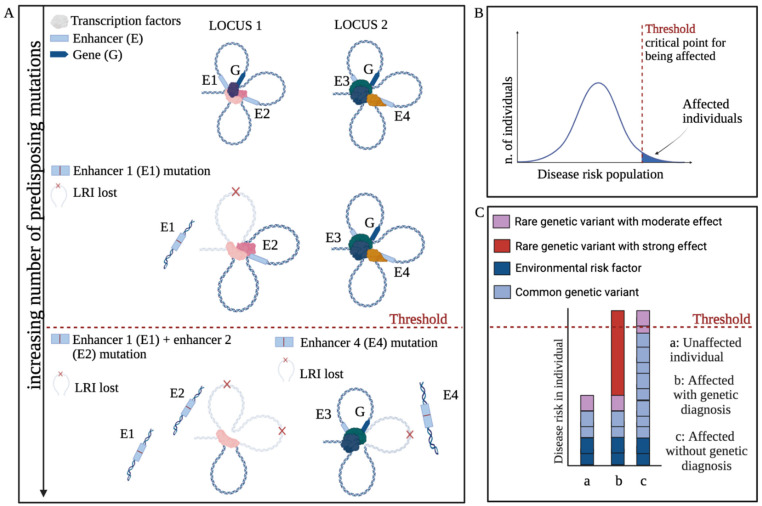
Common and rare mutations build up disease risk via threshold effects. (**A**) The sum of different mutations at enhancers E1, E2, and E4 reaches a threshold for disease predisposition. These mutations may be individually present in normal (non-diseased) individuals (“common” mutations). The same threshold effect may result from quantitative decreases in transcription factors, whose concentration is rate-limiting for the maintenance of the network (see also the discussion in Section 7). Note that a reduction in the efficiency of an enhancer due to mutation may result in reduced gene activation even if the interaction is not completely lost. (**B**) [25] found that “predisposition to rare neurodevelopmental disorders conforms to a threshold liability model, whereby susceptibility to disease is distributed normally in the population; individuals that are beyond a given threshold are affected” (adapted from [26]). (**C**) Different combinations of rare mutations (genetic variants) with stronger effects, and common variants with moderate effects, combined with environmental risk factors, can collectively lead to rare neurodevelopmental disorders. Such mutations may be located in the coding, as well as in the noncoding (regulatory) genome. Note how genotypes of affected (b,c) but also non-affected (a) individuals present with a variety of genetic variants, affecting modes of brain function [25,26] (adapted from [26]).

The analysis by An et al. shows that de novo mutations of such sequences are found in all individuals (probands and controls), suggesting that some mutations of this kind may become part of the “common” mutations pool able to contribute to NDDs not acting by themselves as single mutations, but rather in specific combinations (see Figure 5C). The importance of “common mutations” adding up as small changes that progressively can reach a threshold was recently underscored by the finding that the susceptibility to rare NDDs is distributed normally in the population, with individuals beyond a given threshold becoming affected (see Figure 5B,C) [25].

## 5. Human-Specific Nucleotide Substitutions Within Human Accelerated Regions (HARs) Make Us Different from Our Closest Relatives, the Great Apes

**Figure 6:**
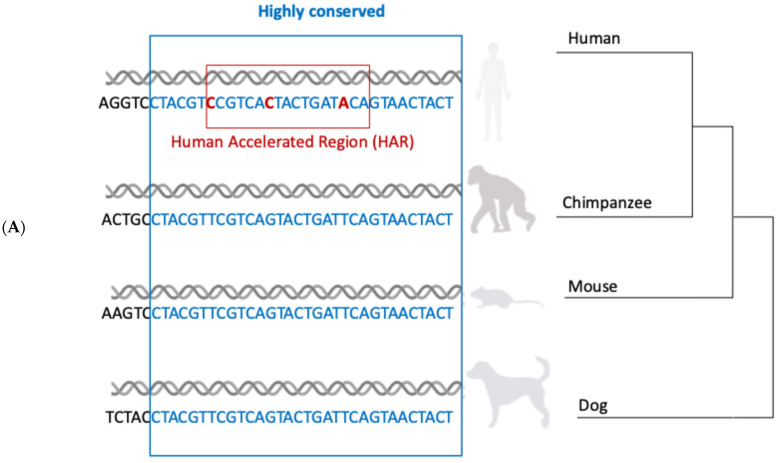

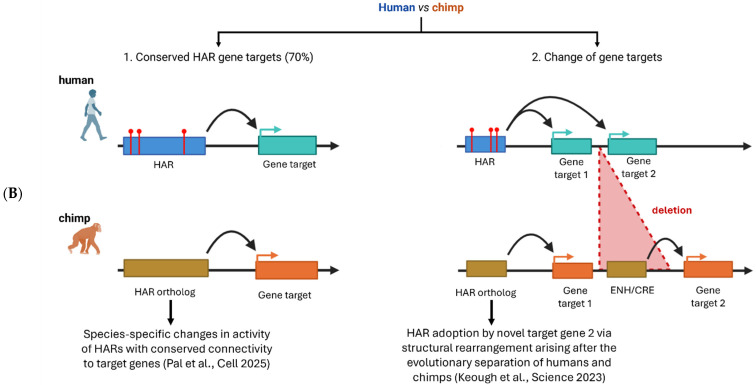
Human-specific nucleotide substitutions within Human Accelerated Regions (HARs) and Human Gained Enhancers (HGE) make us different from our closest relatives, the great apes. Two alternative models explain the evolution of HAR target gene expression. (**A**) HARs are sequences highly conserved in mammalian evolution that have undergone accelerated evolution in humans (i.e., they present with human-specific substitutions, hSubs, in red) (adapted from [11]). (**B**) The two models proposed in [35,36] for the evolution of human-specific HAR-driven gene regulation in the context of the long-range interaction network (see text).

The investigation of the molecular genetic basis of the differences between humans and other species led to the identification of Human Accelerated Regions, noncoding DNA regions that show strong evolutionary conservation, yet contain human-specific nucleotide substitutions (hSubs), arising after the evolutionary split between humans and chimpanzees, our closest relatives, about 6 million years ago (Figure 6A) [11,27,28,29]. These sequences can behave as enhancers in vivo and in vitro, and their activity has been shown to be influenced by many of the identified hSubs in single-gene as well as massively parallel reporter assays (MPRA) [11,30,31,32,33]. Very recently, HARs and their chimpanzee orthologues were functionally studied using single-cell CRISPR interference, leading to the discovery of species-specific gene regulatory functions for some of them [34]. These authors further used prime editing to document differential enhancer activity caused by specific HAR variants; for example, one variant in HAR26;2xHAR.178 was connected to elevated SOCS2 expression and increased neurite outgrowth in human neurons [34]. 

## 6. Two Alternative Models Explain the Evolution of HAR Target Gene Expression, Including ASD-Relevant Genes, from the Point of View of Their Participation in the 3D Interaction Network

Two recent papers addressed the evolution of HARs in relation to the expression of the genes connected to them via long-range interactions, including genes relevant for ASDs and NDDs [35,36] (Figure 6B). Pal et al. [36] experimentally determined interactions for 1590 HARs and 466 human gain enhancers (HGEs) in human and chimpanzee NSCs derived from induced pluripotent stem cells (iPSCs), using Capture HiC (Chi-C), choosing those HARs and HGEs that had differential enhancer activity levels compared to their chimpanzee orthologs. They found that “species differences in HAR and HGE activity were associated with differences in conserved gene target expression, and conserved gene targets (representing 70% of total) were significantly overrepresented among genes showing human-specific expression changes in the brain. By contrast, species-specific targets (30% of total) were not overrepresented” (Figure 6B). They conclude that “HARs influenced brain evolution by altering the expression of ancestral gene targets with neurodevelopmental functions shared between human and chimpanzee rather than by gaining new targets in humans” [36]. Remarkably, they also show that “HAR target gene sets were significantly enriched within gene sets associated with risk for ASD or schizophrenia (SCZ), while HGE targets were not”. Interestingly, HARs were independently proposed to possibly modulate the susceptibility to SCZ, based on available genetic data [37].

Keough et al. [35], on the other hand, highlight the occurrence of structural chromosomal variants (deletions, duplications, translocations) after the split between human and chimp lineages after our latest common ancestor; they propose that structural variants act on gene regulation by causing noncoding regulatory elements, among which HARs, to come in contact with a novel set of genes (Figure 6B). New combinations of noncoding regulatory elements and protein-coding genes would thus be a driving force for the emergence of human-specific traits in this model [35]. It should be noted that the enrichment of HARs in TADs with hSVs is not specific to HARs, as other enhancers are enriched in these TADs as well; further, the available HiC data may not be sufficient to draw final conclusions regarding differences in target genes between human and chimp. It seems likely that this mechanism, though plausibly involved in individual cases, does not represent an alternative global explanation to the one proposed by [36]. Future enhancements in the definition of interaction maps in both species will likely improve our understanding of the relative contribution of the two mechanisms.

## 7. Mutation of a Single Transcription Factor-Encoding Gene, SOX2, Causes Global Changes in the Regulatory DNA Connectivity Network and Global Gene Expression, Affecting the Activity of NDD Genes and HARs

The above observations suggest that the maintenance of the neural 3D interaction network is itself an important function in both NDD and HAR function in brain evolution.

TF mutations are involved in the genesis of many NDDs. Among gene products, whose individual mutations can cause ASDs, TFs are a most frequently mutated category together, along with proteins active at neuron synapses [19,20].

Mutation of the SOX2 TF causes NDDs in both humans and mice [38,39]. Conditional Sox2 mutants in mice uncovered many defects in different regions of the Central Nervous System (CNS), including the brain. At the cellular level, these are rooted in defects of SOX2-expressing NSCs, as well as differentiated neurons and glia: GABAergic neuroblasts, cerebellar Bergmann glia, and thalamic glutamatergic projection neurons connecting the eye to the brain, where SOX2 regulates gene expression together with the TF NR2F1, which is also involved in NDDs [38,40,41,42,43,44].

**Figure 7:**
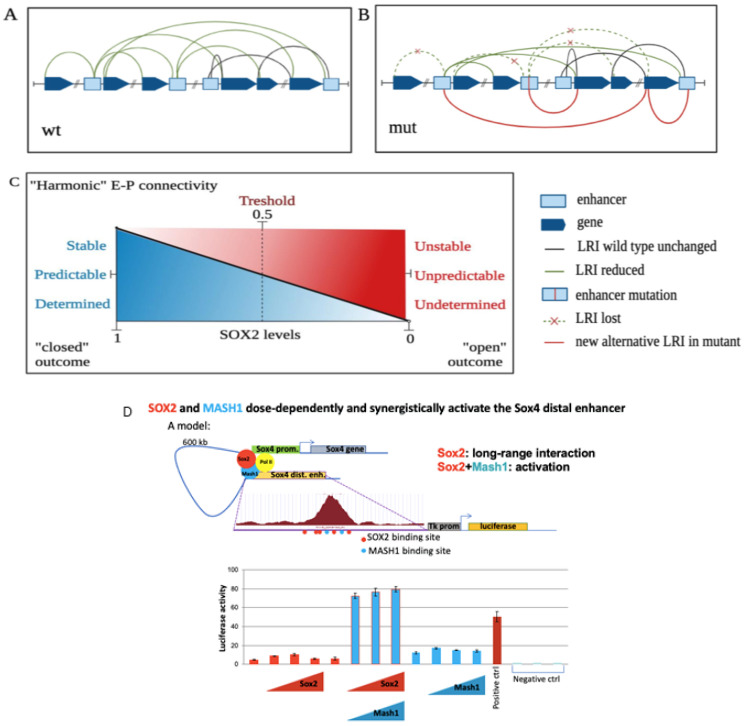
Mutation of a single transcription factor-encoding gene can cause global changes in the regulatory DNA connectivity network and global gene expression. SOX2 is involved in the genome-wide maintenance of the 3D LRI network [3,4]. (**A**) A dense interaction network connects enhancers and gene promoters in brain neural stem cells (**A**). A promoter may be connected to more than one enhancer, and an enhancer to more than one promoter. Promoter–promoter interactions are also observed. (**B**) Following SOX2 loss, this network becomes globally destabilized, contributing to significant changes in the expression levels of about 1000 genes [3]. Many interactions are lost or reduced in frequency; some new interactions are also formed (compare (**B)** with (**A**)). (**C**) The model shown relates TF levels (X axis; SOX2, or possibly YY1) to global patterns of noncoding DNA connectivity, in turn determining gene expression. This might contribute to a more unpredictable, “open” outcome of noncoding DNA interactivity and gene expression in the mutant (**C**) (see Section 10). SOX2 levels = 0.5 correspond to heterozygous mutations in SOX2 (SOX2+/− genotype), causing severe neurodevelopmental disorders in humans. wt: wild type. LRI: long-range interactions. E: enhancers. P: promoters. (**D**) Example: the distal SOX4 enhancer is connected to the SOX4 promoter by a SOX2-dependent interaction [3], and is activated by SOX2 and MASH1 in a dose-dependent manner in transfection assays. Mutations in SOX4 cause NDDs [45].

In mice, genome-wide studies on ex vivo brain-derived neural stem/progenitor cells (NSCs) in Sox2 conditional mutants revealed a profound impairment of the DNA connectivity network; this also favors the manifestation of some novel interactions (mutant-specific), perhaps facilitated by enhancer adoption (Figure 7A,B) [3,4,22].

Of note, transcription factor YY1, whose heterozygous mutation causes Gabriele-de Vries NDD [46,47], is also involved in the maintenance of the LRI network [48] and may contribute to global gene regulation by similar mechanisms.

Loss of just one out of two SOX2 copies causes a neurodevelopmental disorder in humans (though not in mice, [39,42,43], whose molecular mechanisms are still poorly understood. We hypothesize that this involves threshold effects concerning SOX2 binding to dosage-sensitive target regulatory sequences, in turn causing destabilization and reorganization of the interaction network (see model in Figure 7C). It is plausible that, even in the “normal” population, a sub-threshold diversity of SOX2 levels may exist, contributing to differences in the stability of the interaction network between normal individuals.

In the example shown here (Figure 7D), a SOX2-dependent interaction connects a distal enhancer to the promoter of SOX4, a gene whose mutation causes NDDs involving intellectual disability [45]. This enhancer directs the activity of a GFP reporter to the developing zebrafish and mouse forebrain in transgenic experiments [3]. The enhancer is activated by SOX2 in a dose-dependent way, acting synergistically with TF MASH1, co-expressed with SOX2 in the developing ventral forebrain (Figure 7D). Remarkably, the SOX2-dependent connectivity network involves various other genes that are themselves involved in NDDs when mutated, in addition to SOX4 (Table 1). Indeed, many genes whose mutation leads to different types of NDDs are involved in long-range interactions between SOX2-bound promoters and enhancers (Table 1, modified from [3]). Interestingly, when a statistical analysis was performed on classes of genes involved in specific NDDs (as described in the Reviews cited in Table 1), a significant enrichment in SOX2-bound promoter-enhancer interactions was detected (Table 1) [3]; see also Figure 2 in [4].

Future genome-wide studies of gene expression (transcriptomics) and SOX2 binding in human SOX2+/− mutant versus SOX2+/+ cells will identify further SOX2 dosage-sensitive loci at the genome-wide scale to shed light on the general characteristics of the SOX2-orchestrated gene regulatory network in human neural cells and its relevance for neural disease and evolution.

**Figure 8:**
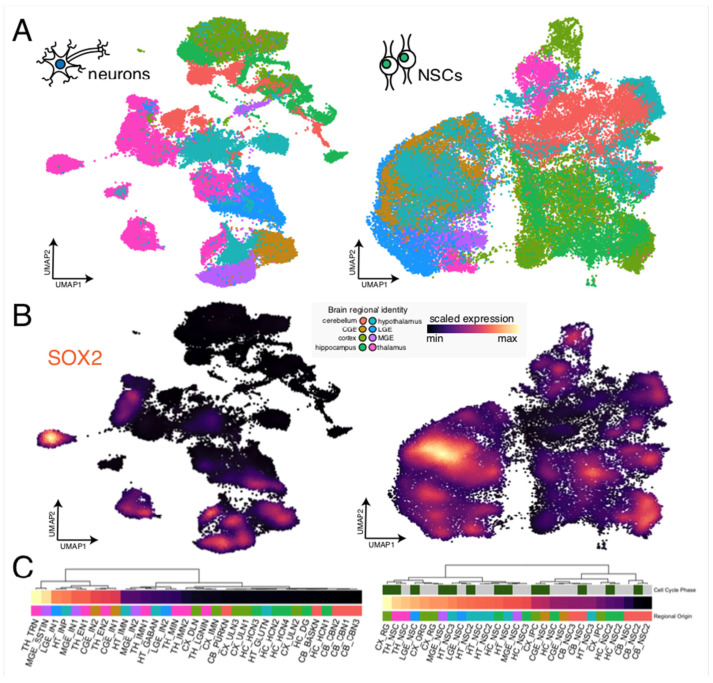
Cell type-specific expression of SOX2 in the prenatal human brain. (**A**) Integrated UMAP representation depicting the transcriptomic similarities of neurons (left) and neural stem cells (NSCs, right) across eight developing human brain regions (cortex, medial ganglionic eminence (MGE), lateral ganglionic eminence (LGE), caudal ganglionic eminence (CGE), hippocampus, thalamus, hypothalamus, and cerebellum) annotated from a publicly available single-cell RNA sequencing (scRNA-seq) atlas [49,50]. (**B**) Density of scaled SOX2 expression overlaid on neuron (left) and NSC (right) cell types in the scRNA-seq atlas. (**C**) Heatmaps depicting average scaled SOX2 expression across 33 neuronal cell types (left) and 29 NSC types (right) in the scRNA-seq atlas. Heatmap annotations show the brain regional origin (bottom) and, for the NSC heatmap, the cell cycle phase (green for G2/M and gray for G1/S) for each cell type.

It will also be interesting to relate SOX2 targets to sites of SOX2 expression within the developing and postnatal human brain. Indeed, available single-cell RNA sequencing (scRNA-seq) atlases [49,50] detect comparatively high levels of SOX2 expression in the thalamus, the hippocampus, and ventral forebrain regions (Figure 8); remarkably, all of these regions were affected following Cre-mediated mutation of mouse Sox2 (see above). Relating changes of gene expression that follow human SOX2 mutation to changes in the abundance of these cell types, as represented in brain organoids, may provide new perspectives to understand the function of the human SOX2 gene.

## 8. Rare, Inherited Mutations Within HARs Are Associated with Autism Spectrum Disorders

**Figure 9:**
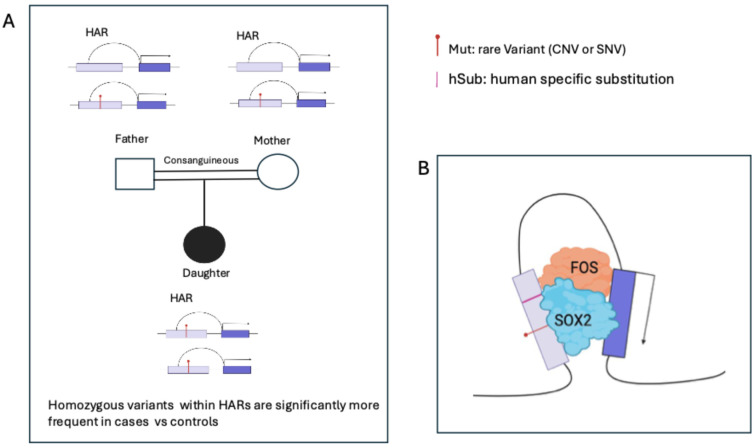

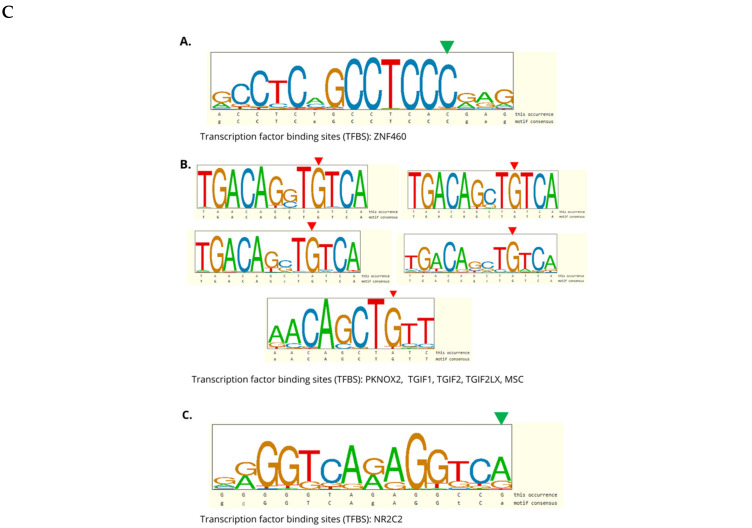
Neurodevelopmental disorders and human-specific evolution are associated with mutations within the same regulatory DNA sequences (HARs). (**A**) Rare recessive DNA sequence variants (CNVs or SNVs) within HARs represent risk factors for autism spectrum disorders [51,52,53]. (**B**) The long-range connectivity of HARs affected by such rare biallelic mutations identifies genes potentially relevant for ASDs. Notably, recognition sites for transcription factor SOX2 are significantly enriched within HARs involved in such interactions; further, SOX2 is also highly enriched among genes involved in such interactions [36,51,52,53]. The function of SOX2 in the maintenance of long-range interactions [3] may play a role in the mechanism whereby these mutations cause disease. HARs (and HGEs) carry human-specific substitutions (hSubs), enriched in binding sites of transcription factor FOS [31], a known SOX2 binding partner in NSC [55]. FOS binding consensus sites are enriched within hSubs, leading to differential enhancer activity in an MPRA assay [31]; SOX2 and FOS together might contribute to human-specific enhancer evolution. (**C**) De novo SNV associated with ASDs (see Figure 4) can overlap single-nucleotide human-specific substitutions and affect transcription factor binding sites (Red or green triangles). In the cases shown, the same nucleotide (triangles above the TF recognition sequences) is affected by disease-associated SNVs [21] and hSubs [31]. Green triangles: the disease-associated SNV converts the human-specific nucleotide into the one found in chimpanzees in the same position. In panel (**C**), A–C show different transcription binding sites affected by different mutations.

A series of recent papers from the Walsh group reported the important finding that rare, inherited, recessive DNA sequence variants (CNV or SNV) located within HARs are enriched in patients with ASDs in consanguineous families, and thus represent “risk factors” for ASDs [51,52,53] (Figure 9). The authors themselves discuss that, while they “find a significant enrichment for rare, recessive variants for HARs (…) in a consanguineous cohort with only 193 probands”, “prior work in non-consanguineous multiple cohorts did not detect a significant contribution of noncoding, inherited variation when examining regions predicted to be functional and suggested that sample sizes of 8000–9000 probands would be required for sufficient statistical power [54]”. Homozygosity is expected to emphasize the effect of enhancer-modulatory mutations (present on both alleles). However, the homozygosity tract may also include other loci, which might contribute to the diseased state by their homozygosity. On the other hand, various ASD-associated variants resulted in altered enhancer activity of the HAR, pointing to their contribution to pathogenesis. The identified HARs were active as enhancers in MPRA assays, and prospective target genes regulated by the HARs were identified by long-range interaction maps (Figure 9). Of note, the authors report a “striking enrichment of SOX2 interactions among genes associated with HARs with rare biallelic mutations”, and that the SOX2 binding site is particularly highly enriched within HARs involved in these interactions (Figure 1H in [51]). Patient-located variants were found to regulate genes that were previously associated with ASDs, but also novel genes that were not, pointing to their possible involvement in pathogenesis [53].

Interestingly, along these lines, a coincidence is observed, at the single nucleotide level, between some human-specific nucleotide substitutions previously mapped to HARs by [31], and SNVs identified in ASD patients by [21], within TF binding consensuses that may thus be implied in brain disease and evolution alike (Figure 9C).

## 9. Coda and Future Directions

Collectively, these findings emphasize that the same noncoding DNA regulatory sequences can act both as HARs, promoting human-specific brain evolution, and as carriers of disease-contributing mutations, involved in mind fragility as seen in NDDs. This yin–yang property of regulatory DNA reflects that of our brain structure, whose great complexity pays its toll with vulnerability.

One present challenge is to connect distal regulatory elements, hosting NDD-relevant sequence variants, to the “right” gene(s) that they regulate. The 3D interaction maps provided by ChIA-PET, HiC, and the like provide hypotheses, but these must be tested by targeted experimental manipulation of the enhancers at their genomic location using CRISPR-based approaches, followed by the assessment of the expression levels of the connected gene [56].

Many genes encoding transcription factors contribute to NDDs through their heterozygous loss-of-function mutations, as discussed for SOX2. How this reflects on their reduced binding to DNA at dosage-sensitive regulatory elements is still to be understood and can be addressed genome-wide, also in small specific neural cell populations, by the sensitive CUT&RUN technology [44,55,57].

We will also have to understand how these mutations result in transcriptional changes, in turn leading to cell diversities. A fruitful (though still expensive) approach to this question is represented by single-cell transcriptomics by RNA sequencing (scRNAseq), to analyze disease-relevant cell populations from normal and mutant genotypes, that can be obtained by in vitro brain organoid development from human pluripotent cells [58,59]. This will allow to define transcriptome-defined cell populations that are selectively vulnerable to dosage reduction of the transcription factor.

A further evolution of the scRNAseq methodologies is represented by Single-Cell Analysis of Five-prime Ends [60], capturing the transcription start sites (TSS). This approach may allow in the future for a definition of differential usage of TSS following TF mutation; this is important in view of the multiple transcription start sites detected in genes involved in NDDs, which may be differentially affected by TF mutation. An example is the 17 TSS of the GPR56 gene, differentially active in the developing dorsal telencephalon; one of them is active in a region containing Broca’s area, involved in language development. This TSS is itself mutated in a disease involving a local abnormality of the cortex (perisylvian polymicrogyria [61].

Finally, WGS revealed a variety of sequence variants in the regulatory genome in “healthy” subjects alike, that, in specific combinations, may result in NDDs. The identification of such combinations needs to be addressed by future studies of WGS in cohorts of patients and their bioinformatic analysis, supported by artificial intelligence (AI), and coupled with functional studies of the effects of the mutations on transcription [62]. “Up close, no one is normal” (“de perto ninguem è normal”, a phrase from a song by Caetano Veloso that is often quoted as it well summarizes the views of psychiatrist Franco Basaglia, whose work led to a profound change in the perspectives and legislation on mental health management in Italy). This seems true also for modern molecular genetics, encouraging us to think of ourselves as a varied bunch of genomes and brains, yet deeply united by our shared humanity.

## 10. Box: Harmonies and Disharmonies, Stabilities and Instabilities, in the Orchestration of Gene Expression, and the Development of New Perspectives for the Mind: A Parallel with Music

By their diverse activity in different individuals, regulative DNA elements collectively contribute to the “harmony” of gene expression orchestration, producing states ranging from an equilibrated, stable brain and mind to more tense, less equilibrated, states, contributing to mind disorder but also, at times simultaneously, to more open and inquiring states of mind (possibly related to traits such as “novelty seeking”) (see Figure 7C).

Regulation of gene expression by noncoding DNA is usually modeled through visual representations (drawings; see Figure 1 and following), but also described by a terminology drawn from music (orchestration, expression, tuning, etc.). We propose to model some of its features by an analogy with music, providing examples that emphasize the importance, for specific expression features, of specific combinations of notes within a scale or a chord, and of the dynamic connection of chords within a phrase (see also Figure 1C). We highlight how small but important changes in the note composition of a chord and the dynamics of the interactions between chords can result in big changes in the global emotional significance of the piece, changing it from harmonic to disharmonic, from peaceful to tense, or vice versa (compare to Figure 7C). We note, in this respect, how a change felt as “disharmonic”, unexpected to already known, well-established harmonies, may at the same time be an exploration of new avenues of expression, and contribute a novel emotional perspective to the piece (see examples below). This might parallel the function of some combinations of noncoding DNA elements, leading to increased frailty and vulnerability to distress in the individuals carrying them, but at the same time conferring increased potential for invention and exploration regarding new ways of thought and creativity. Furthermore, the importance of the time dimension in music reminds us of its importance for neural gene regulation, developing dynamically in the course of embryogenesis. This parallel between music and molecular genetics, both dealing with invisible entities (music and DNA), might help to develop our imagination along novel perspectives.

1. Beethoven—Sonata Op. 111 No. 32 in C minor-1

Traveling through a beloved, well-known landscape to explore new aspects of it and then returning back home along a known horizon:

https://www.youtube.com/watch?v=WGg9cE-ceso from time 9.20 to 10.30, accessed on 15 January 2026.

2. Beethoven—Sonata Op. 111 No. 32 in C minor-2

Exploring uphill to reach a mountain pass that opens into unknown landscapes. Several ways are possible and our choice is undetermined, and suspended into the unknown:

https://www.youtube.com/watch?v=WGg9cE-ceso from time 13.58 to 14.58, accessed on 15 January 2026.

3. Liszt-Bagatelle sans tonalité

The Bagatelle rhythm is still easily recognized, but the reference to the “home feeling” of classical tonality is lost:

https://www.youtube.com/watch?v=yc_HjEa8k5k, accessed on 15 January 2026.

4. Shoenberg—Sechs kleine Klavierstuecke op. 19

Exploring new connections between notes, and between notes and feeling, in the world of atonality—a novel, unfamiliar yet intriguing world for the brain:

https://www.youtube.com/watch?v=TZleqbjwEuA, accessed on 15 January 2026.

## Figures and Tables

**Table 1:** SOX2 binds to enhancers and connected gene promoters of multiple genes whose mutations contribute to NDDs. The table lists genes involved in the indicated NDDs in humans (“Category of disease” column), which have phenotypic overlap with the disorders observed in SOX2-mutant patients (hippocampal hypoplasia, eye defects; intellectual disability and seizures) and/or Sox2-mutant mice (microcephaly, hippocampal defects, seizures, eye defects). The dots indicate the presence of SOX2 binding, in the respective mouse gene promoter (right column: Disease gene promoter, SOX2-bound), or a distal enhancer interacting with that promoter (“Disease gene promoter, interaction with SOX2-bound enhancer” column), in RNApolII-ChIA-PET interaction datasets obtained in mouse brain-derived neural stem cells. The indicated *p*-values are based on the comparison between the genes involved in SOX2-bound promoter–enhancer interactions and the total number of genes within groups of genes relevant for each NDD, taken from the indicated Review references at the bottom of Table 1 (modified from [3], Table S6).

Category of Disease		The Disease Gene Promoter		
Mouse Gene Name	Human Gene Name	Interacts with a Distal Enhancer	Interacts with a SOX2-Bound Enhancer	Is SOX2-Bound
**Microephaly associated to defects in** **1) Centrosome and spindle microtubule (1)** Positive: 10/22; *p*-value: 0.006				
Cdk5rap2 (Mcph3)	*CDK5RAP2*			•
Casc5	*KNL1*			•
Cenpj	*CENPJ*			•
Stil	*STIL*			•
Cep63	*CEP63*	•	•	
Kif2a	*KIF2A*	•		
Kif11	*KIF11*	•		
Tubb2b	*TUBB2B*	•	•	•
Tuba1a	*TUBA1A*	•	•	•
Poc1a	*POC1A*			•
**2) Origin recognition complex core (1)** Positive: 2/5				
Orc4	*ORC*			•
Cdt1	*CDT1*			•
**3) DNA damage response and repair (1)** Positive: 5/19				
Lig4	*LIG4*			•
Phc1 (Mcph11)	*PHC1*	•	•	•
Xrcc2	*XRCC2*			•
Xrcc4	*XRCC4*			•
Blm (Recql3)	*BLM*			•
**Other microcephalies**				
Gpr56	*ADGRG1*	•	•	•
Cdk19	*CDK19*			•
Arx	*ARX*	•		
Zbtb18	*ZBTB1B*			•
**Angelman and Angelman-like syndromes (2) (intellectual disability and absent speech)** Positive: 10/12; *p*-value: 0.0000065				
Ube3a	*UBE3A*			•
Tcf4	*TCF4*	•	•	•
Ehmt1	*EHMT1*			•
Herc2	*HERC2*	•	•	
Adsl	*ADSL*			•
Cdkl5	*CDKL5*			•
MeCP2	*MECP2*			•
Foxg1	*FOXG1*	•	•	
Atrx	*ATRX*			•
Zeb2	*ZEB2*	•	•	•
**Histone modification, chromatin remodelling and mediator mutations (3,4,5)** Positive: 8/12; *p*-value: 0.0007				
Med17	*MED17*			•
Med23	*MED23*			•
Med25	*MED25*			•
Smarca2	*SMARCA2*			•
Arid1a	*ARID1A*	•	•	
Arid1b	*ARID1B*	•		
Jmjd1c	*JMJD1C*	•	•	
Phf21a	*PHF21A*			•
**Cohesin subunit mutations (6) (psychomotor delay, intellectual disability)** Positive: 4/14				
Smc3	*SMC3*			•
Rad21 (Scc1)	*RAD21*			•
Stag1	*STAG1*	•	•	
Stag2	*STAG2*	•		
**Microphtalmia/Anophtalmia/Coloboma and other eye pathologies (7)**				
Otx2	*OTX2*	•		•
Pax6	*PAX6*			•
Six3	*SIX3*			•
Bmp7	*BMP7*	•	•	
Grcc10 (C12orf57)	*C12orf57*	•		•
Sall2	*SALL2*			•
RarB	*RARB*	•		•
Smoc1	*SMOC1*	•	•	
Wdr19	*WDR19*			•
COUP-TF1 (Nr2f1)	*NR2F1*	•	•	
Abhd12	*ABHD12*	•	•	
**References:**				
(1) Alcantara and O’Driscoll, 2014, AM J Med Genet C Semin Med Genet 166C, 124				
(2) Tan et al., 2014, Am J Med Genet A 164, 975–992				
(3) Lee and Young, 2013, Cell 152 1237–1251				
(4) Saez et al., 2016, Genetics in Medicine 18, 378–385				
(5) Kim et al., 2012, Am J Hum Genet 97, 56–72				
(6) Peters et al., 2008, Genes Dev 22, 3089–3114				
(7) Williamson and Fitzpatrick, 2014, Eur J Med Genet 57, 369–380				

## Data Availability

The raw data supporting the conclusions of this article will be made available by the authors on request.
